# Is the Canadian legal framework too restrictive and based on false premises?

**DOI:** 10.1186/2054-3581-1-8

**Published:** 2014-05-29

**Authors:** Marie-Chantal Fortin

**Affiliations:** Centre de recherche du Centre hospitalier de l’Université de Montréal (CHUM), Montreal, Canada; Bioethics Program, Department of Social and Preventive Medicine, École de santé publique de l’Université de Montréal, Montreal, Canada; Nephrology and Transplantation Division, Centre hospitalier de l’Université de Montréal (CHUM), 1560 Sherbrooke Street East, Montreal, Quebec H2L 4M1 Canada

## Introduction

Given the increasing shortage of organs in recent years, there has been an abundant empirical and theoretical literature on incentives to improve organ donation [1, 2]. Some of these incentives, such as reimbursement of donor expenses, are uncontroversial whereas others, such as payment for organs, are matters of heated debate. In this issue of the Journal, Caulfield and colleagues provide an in-depth legal analysis of various incentives in the Canadian context [3]. They show us that in Canada, organ donation is based on altruism; organs are viewed as a gift; and the law dictates that organ donation should be “gratuitous” and without any “rewards and benefits” or “valuable consideration”. This means there can be no measures that might encourage or influence organ donation. As the authors demonstrate, this legal framework is restrictive and bans not only the trade of organs, but also most of the monetary and non-monetary incentives currently used in organ donation. In this editorial, I will discuss the relevance of the principle of altruism in organ donation and the contradiction between the legal definition of a gift and gift-exchange theory and show how the current Canadian legal framework is out of sync with the empirical data on incentives.

### Altruism and organ donation

Altruism is a key principle in our current organ donation and transplantation legislation, and is also a matter of scholarly debate in the philosophical, sociological and psychological literature [4–6]. Following are some examples of unanswered questions regarding the nature of altruism:

- Do motivations make an action altruistic?
- Is there such a thing as pure altruism? If so, most living organ donors are not altruistic, since they have mixed motivations [7, 8].
- Should an altruistic act be directed toward strangers or can it be directed toward loved ones? If this is the case, only anonymous non-directed donation would be altruistic.
- Finally, can altruistic agents benefit from their gesture? If not, living organ donors who experience increased self-esteem and other psychological benefits cannot be considered altruistic.

As shown in a previous study, altruism holds a different meaning for transplant professionals who describe a wide variety of altruistic actions, ranging from acts of common courtesy to heroic actions such as saving lives [9]. Incentives and even the removal of disincentives to organ donation could be considered incompatible with the idea of pure altruism. I concur with other authors who find it problematic for organ donation to be based on this vague and debated concept of altruism [10]. As proposed by Saunders, solidarity might be a better guiding principle [11].

### Gift exchange theory

In their review, Caulfield et al. report that the legal prohibition of any incentives in organ donation is tied to its definition as a gift [3]. The legal definition of a gift does not concur with anthropological theories of gift exchange. In his seminal essay on the gift in traditional societies, Marcel Mauss described a triple set of obligations: to give, receive and repay. Individuals who refuse one of these obligations create social tension [12]. Renée C. Fox and Judith Swazey have shown that Mauss’ obligations are present in both deceased and living organ donation [13]. In organ transplantation, it is not only the patient who receives a gift, but also society at large [6]. This is more obvious in the case of deceased organ donation, where the organ procurement organization receives the organ and allocates it to a patient. In living organ donation, society likewise benefits, since the gift allows citizens to live active, fulfilling lives, to pay taxes, and so on. Society further benefits from the donation, since hemodialysis is far more expensive than kidney transplantation [14]. If society is also a recipient of this gift, it has an obligation to repay the donor (or the donor’s family in the case of deceased organ donation). Incentives such as recipient priority in exchange for donating, donor recognition events, payment of funeral expenses and tax credits could easily be envisioned as a way for society to repay such a gift.

### Empirical evidence and incentives

As mentioned by Caulfield et al., Canadian laws are out of step with current practices and public perspectives on incentives in organ donation [3]. A recent survey of the Canadian public, health professionals and patients revealed a high acceptance of incentives such as reimbursement of funeral expenses, tax breaks or credits for deceased donation, and reimbursement of donation-related expenses and lost wages in the case of living organ donation [1]. Further, it is current practice in many Canadian provinces to reimburse expenses related to living organ donation [15–17]. In another recent study conducted in Europe, North America, Australia and New Zealand, transplant professionals found the reimbursement of expenses associated with living organ donation to be acceptable. Some even considered a regulated organ market to be acceptable [2]. Israel has seen an increase in organ donation rates since passing a law in 2009, which gives priority points to organ donors and their families, and removes disincentives for living donors [18].

## Conclusion

Caulfield et al.’s legal review demonstrates that the federal and provincial legal framework in Canada is too restrictive for incentives related to organ donation, and that it is based on a poorly conceptualized view of altruism (and its limits) [3]. Considering well-accepted anthropological theories on the gift exchange, it is contradictory for organs to be conceived in a narrow legal sense, thus precluding any form of reciprocity. The current legal framework does not capture the complex reality of organ donation, whether living or deceased. Perversely, an overly restrictive legal framework that makes some incentives for organ donation illegal could actually encourage unethical practices, such as transplant tourism, where desperate patients travel abroad to be transplanted because there are insufficient organs available at home. Finally, incentives for organ donation are just one example of how lawmakers are lagging behind social changes and practices (cf. the debate on physician-assisted suicide).
